# Planting Density Affects *Panax notoginseng* Growth and Ginsenoside Accumulation by Balancing Primary and Secondary Metabolism

**DOI:** 10.3389/fpls.2021.628294

**Published:** 2021-04-12

**Authors:** Haijiao Liu, Hongrui Gu, Chen Ye, Cunwu Guo, Yifan Zhu, Huichuan Huang, Yixiang Liu, Xiahong He, Min Yang, Shusheng Zhu

**Affiliations:** ^1^State Key Laboratory for Conservation and Utilization of Bio-Resources in Yunnan, Yunnan Agricultural University, Kunming, China; ^2^Key Laboratory for Agro-Biodiversity and Pest Control of Ministry of Education, Yunnan Agricultural University, Kunming, China; ^3^School of Landscape and Horticulture, Southwest Forestry University, Kunming, China

**Keywords:** ginsenosides, *Panax notoginseng*, plant density, primary metabolism, secondary metabolism

## Abstract

Adjusting planting density is a common agricultural practice used to achieve maximum yields. However, whether the quality of medicinal herbs can be improved by implementing appropriate planting densities is still uncertain. The medicinal crop *Panax notoginseng* was used to analyze the effects of planting density on growth and ginsenoside accumulation, and the possible mechanisms of these effects were revealed through metabonomics. The results showed that *P. notoginseng* achieved high ginsenoside accumulation at high planting densities (8 × 8 and 10 × 10 cm), while simultaneously achieved high biomass and ginsenoside accumulation at moderate planting density of 15 × 15 cm. At the moderate planting density, the primary metabolism (starch and sucrose metabolism) and secondary metabolism (the biosynthesis of phytohormone IAA and ginsenoside) of the plants were significantly enhanced. However, the strong intraspecific competition at the high planting densities resulted in stress as well as the accumulation of phytohormones (SA and JA), antioxidants (gentiobiose, oxalic acid, dehydroascorbic acid) and other stress resistance-related metabolites. Interestingly, the dry biomass and ginsenoside content were significantly lower at low densities (20 × 20 and 30 × 30 cm) with low intraspecific competition, which disturbed normal carbohydrate metabolism by upregulating galactose metabolism. In summary, an appropriate planting density was benefit for the growth and accumulation of ginsenosides in *P. notoginseng* by balancing primary metabolism and secondary metabolism.

## Introduction

Medicinal herbs, which can be used directly for therapeutic purposes or as important resources for pharmacological drug research and development, have a long history and play an indispensable role in the prevention and treatment of many diseases (Liu et al., 2015). The pharmacological effects of medicinal herbs depend on the contents of their various secondary metabolites, including alkaloids, terpenes, phenols, fatty acids, and fatty oils (Yang et al., 2018a). Many factors influence the accumulation of secondary metabolites (Kim et al., 2015). Secondary metabolites are primarily regulated by the primary metabolic pathways (Caretto et al., 2015), which provide accessible energy for normal plant growth and carbon skeletons for the biosynthesis of secondary metabolites (Ramakrishna and Ravishankar, 2011; Caretto et al., 2015; Liu et al., 2017a). Additionally, the accumulation of secondary metabolites is regulated by biotic and abiotic stresses (Guo et al., 2020). Importantly, the intensity of such stresses affects the balance between the primary and secondary metabolism, which subsequently affects the growth, development and quality of genuine herbal medicine. Moderate stress promotes the accumulation of secondary metabolites in medicinal plants (Yang et al., 2018a; Guo et al., 2020). However, excessive stress restricts resource acquisition, which inevitably affects the growth of plants then ultimately affects the accumulation of secondary metabolites (Huang and Guo, 2007; Guo et al., 2020). Therefore, the utilization of a suitable stress level to balance the primary and secondary metabolism is a critical strategy for achieving both high yields and high qualities of medicinal herbs simultaneously.

*Panax* plants (such as *P. notoginseng, P. ginseng* and *P. quinquefolius*), have been used for nearly 5,000 years in Oriental medicine and more recently in Western medicine (Kim et al., 2015). Among the compounds in *Panax* plants, ginsenosides are the main active pharmaceutical components and have shown multiple medicinal effects, such as antioxidative, antiaging, and anticancer effects as well as other health-improving activities (Mancuso and Santangelo, 2017; Zhang S. et al., 2018; Zhou et al., 2019). Ginsenosides in *P. notoginseng* are synthesized via the mevalonic acid pathway, which uses the intermediate product of primary metabolism, acetyl-CoA, to generate the precursor 2,3-oxidosqualene and then forms triterpenoid ginsenosides (Ghosh, 2016; Chen et al., 2017; Li et al., 2019). Thus, changes in the primary metabolism have been shown to affect the accumulation of ginsenosides in *P. ginseng* and *P. quinquefolius* (Liu et al., 2017a,b). In addition, previous studies have demonstrated that ginsenoside content is also affected by the species and developmental stages of the plant (Kim et al., 2015) as well as by various agricultural practices, including fertilization, soil water, light availability, etc. (Kim et al., 2012; Wei et al., 2018; Zhang T. et al., 2018). Of these influencing factors, agricultural practices are the most economic and effective management technique for improving the accumulation of ginsenosides. Many previous studies have shown that intraspecific interactions can affect the morphological characteristics of the plant canopy (Song et al., 2016) and roots (Khan et al., 2018) as well as the plant metabolic level (Kuai et al., 2016), ultimately influencing plant growth and quality (Huang et al., 2019). The effects of intraspecific interactions on plant performance can be easily realized by altering the planting density (Broekman et al., 2019; Ning et al., 2019). A suitable density ensures the normal growth of individual plants and entire population through coordinate utilization of water, light, temperature, and nutrients (Ning et al., 2019). However, an overly high planting density results in stress and low resource availability, eventually decreasing the accumulation of secondary metabolites (Bascuñán-Godoy et al., 2016; Ors and Suarez, 2017). Thus, it is important to balance the primary and secondary metabolism by adjusting the plant density. Whether the growth and quality of *Panax* can be improved by creating appropriate stress by regulating the planting density is worthy of research.

*P. notoginseng* is a famous traditional Chinese medicine with therapeutic effect on cardiovascular diseases (Yang et al., 2019). Previous studies suggested that (10~15) × (12~20) cm was the commonly used planting density of *P. notoginseng* seedlings, which were determined mainly on the basis of yield and economic benefits (Cui et al., 1998; Zheng et al., 2015; Ou et al., 2018; Li et al., 2020). In this study, five planting densities, including 8 × 8, 10 × 10, 15 × 15, 20 × 20, and 30 × 30 cm, were set to test the effects of plant density on (1) seedling growth parameters, root architecture and root phytohormone content; (2) the content of ginsenosides, determined by UPLC; and (3) the contents of primary and secondary metabolites, determined by GC-MS. Then, we further analyzed the correlations between ginsenoside accumulation and primary and secondary metabolites under intraspecific interaction conditions. Based on these analyses, we expected to found a suitable plant density for achieving both high yield and high qualities of medicinal herbs simultaneously.

## Materials and Methods

### Experimental Design

**Figure 1:**
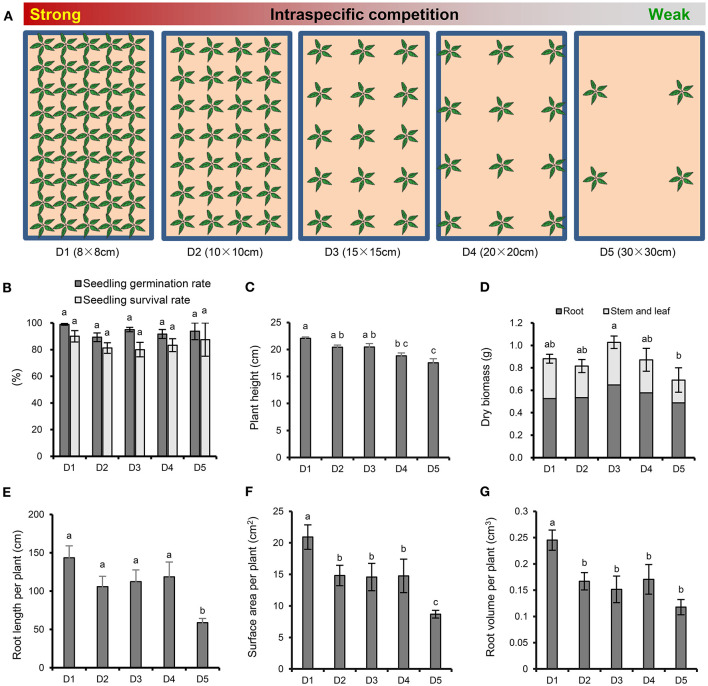
Experimental scheme and effects of plant density on the growth and root morphology of *P. notoginseng*. **(A)** Experimental design with five *P. notoginseng* planting densities. D1~D5 represent densities of 8 × 8, 10 × 10, 15 × 15, 20 × 20, and 30 × 30 cm, respectively. **(B)** Seedling germination rate and survival rate. **(C)** Plant height. **(D)** Dry biomass. **(E)** Root length per plant. **(F)** Surface area per plant. **(G)** Root volume per plant. Error bars indicate the standard error (SE). Different letters indicate statistically significant differences among different treatments (*p* < 0.05; *n* = 4).

The experimental design for *P. notoginseng* cultivation is shown in Figure 1A. Each plastic basin (65 × 40 × 18 cm) contains about 40 kg natural soil, which was collected from a pine forest in Xundian Country, Yunnan, China (103.29°E, 25.51°N; altitude of 1,960 m), then sieved to remove the residue of plant. The soil had the following characteristics: pH 5.17, electrical conductivity 458 μS cm^−1^, available potassium (K) 6.90 mg kg^−1^, available phosphate (P) 5.18 mg kg^−1^, alkali-hydrolyzable nitrogen (N) 172.38 mg kg^−1^ and organic matter 47,830 mg kg^−1^. A total of 4, 12, 15, 28, 45 healthy one-year old seedlings were planted in January 3, 2016 at a plant spacing of 30, 20, 15, 10, and 8 cm, respectively. There were four repeats for each treatment, and a total of 20 plastic basins were placed in a completely randomized block design in greenhouse. The emergence rate of *P. notoginseng* was recorded in March 20, 2016, and the survival rate was recorded in September 17, 2016. All plants were harvested to measure the plant height and dry biomass (roots, stems and leaves) were measured in November 30, 2016. In addition, the architecture of the fresh fibrous roots was analyzed using an optical scanner (Epson Perfection V850 Pro), and the average root length, surface area and root volume per plant were analyzed with image analysis software (WinRHIZO Tron MF, Regent).

### Determination of the Phytohormone Content in *P. notoginseng* Roots

Changes in the contents of jasmonic acid (JA), auxin (IAA), salicylic acid (SA), and abscisic acid (ABA) in *P. notoginseng* fibrous roots were determined by HPLC-MS/MS according to Fu's method (Fu et al., 2012). The fibrous roots were collected in October 2016. Each treatment contained four replicates. Approximately 0.3 g fibrous roots was transferred to FastPrep tube, which containing 0.9 g of FastPrep matrix, then flash-frozen in liquid nitrogen and stored at−80°C. A total of one milliliter of acetate spiked with 200 ng of internal standards (^13^C_2_-JA, D_4_-SA, D_5_-IAA and D_6_-ABA) were added to each sample and homogenized. After 10 min centrifugation at speed of 12,000 g at 4°C, the supernatants were moved to fresh Eppendorf tubes. Each sample was re-extracted with 0.5 mL of ethyl acetate and centrifuged; the supernatants were combined and then evaporated on a vacuum concentrator. The residue was resuspended by 0.5 mL of 70% methanol (v/v) and centrifuged to clarify the phases. The supernatants were transferred to autosampler vials for further analysis. A total of 15 μL of each sample was injected into a Pursuit C8 column (3 μm, 150 mm × 2 mm) (Varian) with a flow rate at 0.1 mL min^−1^. Solvent A (0.05% formic acid) and solvent B (methanol) was used as mobile phase. Negative electrospray ionization mode was used for detection. Each phytohormone was quantified by comparing its peak area with the peak area of its respective internal standard.

### Ginsenosides Extraction and UPLC Analysis

According to Yang's method (Yang et al., 2015), 0.2 g of powdered *P. notoginseng* taproots or fibrous roots was transferred into a 50 mL tube and ultrasonically extracted with 15 mL 70% MeOH (v:v) at 25°C for 30 min. After centrifuged at 12,000 g for 5min, the supernatants were filtered with a 0.22 μm nylon membrane filter and collected at 4°C for further analysis. Each treatment included four replicates. The ginsenosides were quantified using a Nexera X2 UPLC system (Shimadzu, Japan) equipped with a diode array detector (DAD) and a Poroshell 120 EC-C_18_ reversed-phase column (150 × 4.6 mm, 4 μm, Agilent). The initial injection volume was 10 μL and the flow rate was 1.0 mL min^−1^. A solvent system consisted of the linear gradient of solvent A (acetonitrile) and solvent B (0.1% phosphoric acid in water) was used for separations (Supplementary Table 1). The column temperature was maintained at 30°C. Chromatograms were recorded at 203 nm. The ginsenosides, including Rg_1_, Rb_1_, R_1_, Re, and Rd, in the samples were identified and quantified by comparing the retention time and peak areas to authentic ginsenoside standards (Supplementary Figure 1).

### Analysis of the Relative Expression Level of Dammarenediol-II Synthase (*DS*)

The relative expression level of *DS*, the first committed step in the synthesis of dammarene-type triterpenoid ginsenosides (Li et al., 2019), was quantified by Real-time PCR. Total RNA was isolated from fibrous roots of *P. notoginseng* using *TransZol*™ Up Plus RNA kit (ER501, TransGen Biotech). First-strand cDNA was synthesized with 100 ng total RNA using a TransScript miRNA First-strand cDNA synthesis SuperMix kit (AT351-01, TransGen Biotech). Real-time PCR was performed with a LightCycler 96 (Roche) and SYBR Green Reagents (Bio-Rad) using gene-specific primers and the following cycle conditions: 2 min at 95°C, and 40 cycles of 15 s at 95°C, 15 s at 52°C and 60 s at 72°C. The genes expression was normalized by the internal control *18S rRNA* and defined the treatment of 15 × 15 cm as the control group. The level of gene expression was calculated using the 2^−Δ*ΔCt*^ method (Han et al., 2020). All primers used in this study were listed in Supplementary Table 2.

## Determination of Metabolites in Fibrous Roots

### Sample Preparation

Approximately 60 mg of frozen powder fibrous roots and 1 mL of methanol (CH_3_OH) containing 0.5 mg of ribitol (internal standard, Supplementary Figure 2) were added into a prechilled 2 mL lock-cap centrifuge tube, then vortexed for 10 s. A 300 μL extraction aliquot (H_2_O:methanol:chloroform = 1:2.5:1, v:v:v) was added and ultrasonically extracted for 30 min at 37°C. Then, the sample was centrifuged (1,600 g, 3 min) to separate the polar and nonpolar phases. Then the upper polar phase was transferred to a fresh centrifuge tube and added 200 μL sterile water, and then vortexed and centrifuged (1,600 g, 4°C for 3 min). A 250 μL aliquot of the upper phase was transferred to a fresh centrifuge tube, dried for 3–4 h at room temperature using a SpeedVac (Christ, Germany). Adding 80 μL of methoxyamine hydrochloride solution (20 mg mL^−1^ dissolved in pyridine) to each sample and incubating for 90 min at 30°C to protect carbonyl moieties. And then, 40 μL N-methyl-N-(trimethylsilyl)-trifluoroacetamide (MSTFA) was added and incubating at 37°C for 30 min to trimethylsilylate the acidic protons. After this step, the sample was centrifuged (1,600 g, 4°C for 3 min), then the supernatant was stored at 4°C for further analysis.

### GC-MS Analysis

GC-MS analysis was done according to previous method (Robinson et al., 2007) using a Shimadzu QP 2010 instrument equipped with an AOC-5000 autosampler (Shimadzu, Japan) and a SH-Rxi-5Sil MS capillary column (30 m × 0.25 mm × 0.25 μm, Agilent). Helium (99.99% purity) was used as the carrier gas at a flow rate of 1.0 mL min^−1^. The injection volume was 0.8 μL in split mode (10:1), and the injector and transfer line temperature were 280°C. The initial column temperature was 100°C (held for 4 min) and was programmed to increase at a rate of 4°C min^−1^ to 320°C (held for 8 min). Mass spectra were obtained in electron impact (EI) ionization mode at 70 eV by monitoring the full-scan range (m/z 45-600). The raw data of metabolites can be found with the URL www.ebi.ac.uk/metabolights/MTBLS2634 and the accession number is MTBLS2634.

### Metabolite Profiling Analysis

Acoording to Ji's method (Ji et al., 2017), the raw peak obtained by data baseline filtering and calibration, peak alignment, deconvolution analysis and peak identification using MS-DIAL with the Fiehn library and the identification parameters were list in the Supplementary Table 3. The peak areas of metabolites in raw MS-DIAL output (Supplementary Table 4) were normalization by sum, transformation by log and scaling by Pareto method on Metaboanalyst 4.0 (http://www.metaboanalyst.ca/MetaboAnalyst/) (Chong et al., 2019). Simca-P 14+ was used for the principal component analysis (PCA) and orthogonal projection to latent structures-discriminant analysis (OPLS-DA). The differentially accumulation metabolites (DAMs) were screened for variable importance in the projection (VIP) >1 and *p* < 0.05 and then mapped to the biological pathways in the Kyoto Encyclopedia of Genes and Genomes (KEGG) database.

### Statistical Analysis

SPSS 18.0 software (SPSS Inc., USA) and Origin 8.0 (OriginLab Inc., USA) were used for analyzing the raw data and graphing. One-way analysis of variance (ANOVA) with Duncan's multiple range test, *t*-tests and correlation analysis with the Pearson correlation coefficient were used in the data analysis.

## Results

### Effects of Plant Density on *P. notoginseng* Growth

There were no significant differences among the different densities in the emergence and survival rates of *P. notoginseng* (Figure 1B). With the increasing strength of the interspecific interaction, the plant height (Figure 1C) and the root architecture, including the root length (Figure 1E), surface area (Figure 1F) and root volume (Figure 1G), showed an increasing trend. In particular, the strongest interspecific interaction (8 × 8 cm) resulted in a significant increase in plant height, root length, surface area and root volume compared with the weakest interspecific interaction (30 × 30 cm). It is worth noting that the whole-plant, root, and aboveground dry biomasses were higher at density moderate of 15 × 15 cm than at the high and low densities (Figure 1D). The whole-plant dry biomass was significantly lower when *P. notoginseng* was cultivated at the lowest density (30 × 30 cm) than at D3 (15 × 15 cm).

### Effects of Plant Density on the Phytohormone Content in Roots

**Figure 2:**
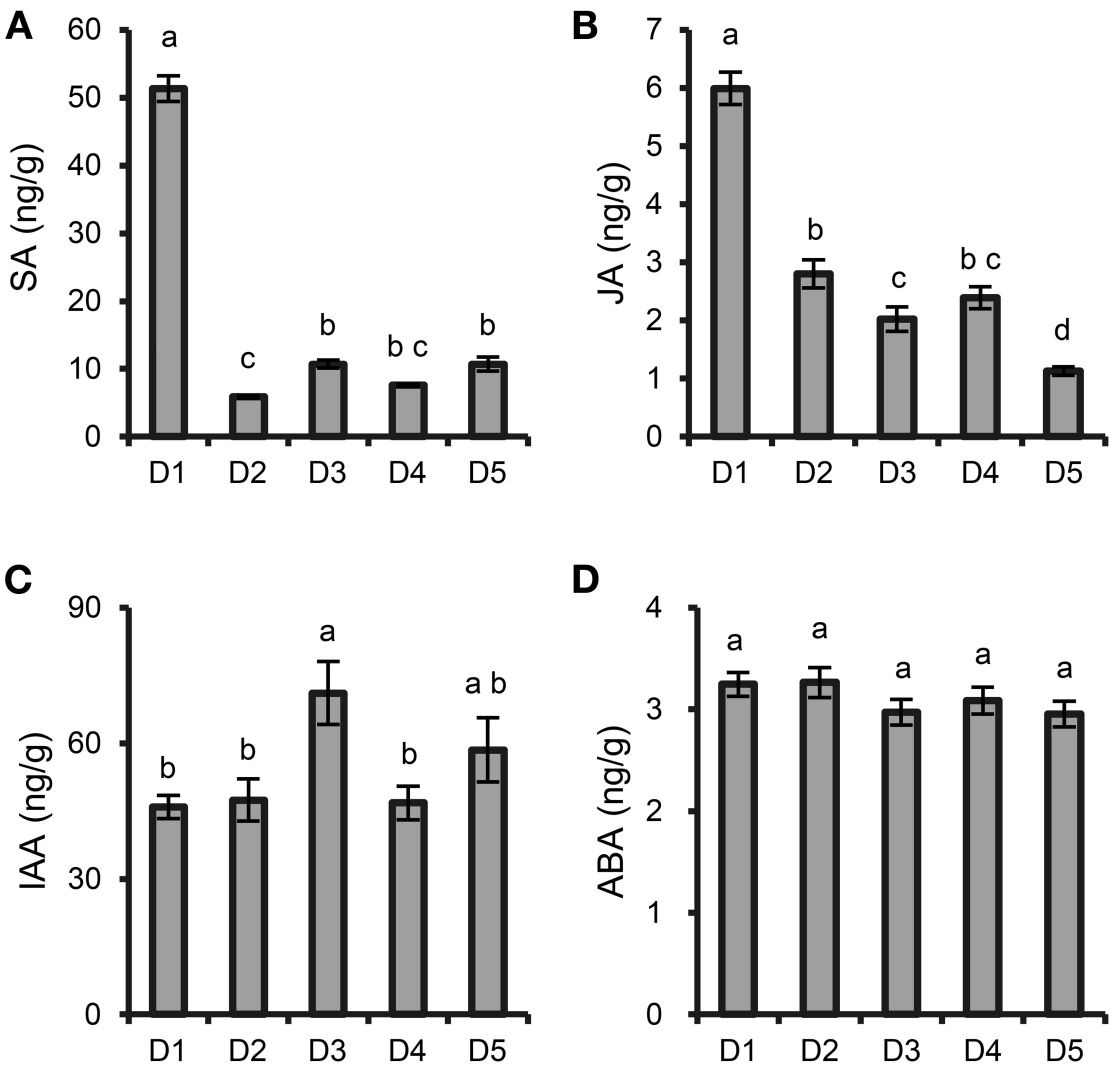
Effects of plant density on phytohormone content in *P. notoginseng* roots. **(A)** SA. **(B)** JA. **(C)** IAA. **(D)** ABA. D1-D5 represent *P. notoginseng* grown at densities of 8 × 8, 10 × 10, 15 × 15, 20 × 20, and 30 × 30 cm, respectively. All data are presented as the mean ± standard error (SE). Different letters indicate statistically significant differences among different treatments (*p* < 0.05; *n* = 4).

The contents of SA and JA were significantly increased at the highest density (8 × 8 cm) compared with those at the other densities (Figures 2A,B). The content of IAA at moderate density (15 × 15 cm) was higher than those at the other densities (Figure 2C), while the content of ABA was not significantly different among the different densities (Figure 2D).

### Effects of Plant Density on the Ginsenoside Content and *DS* Expression

**Figure 3:**
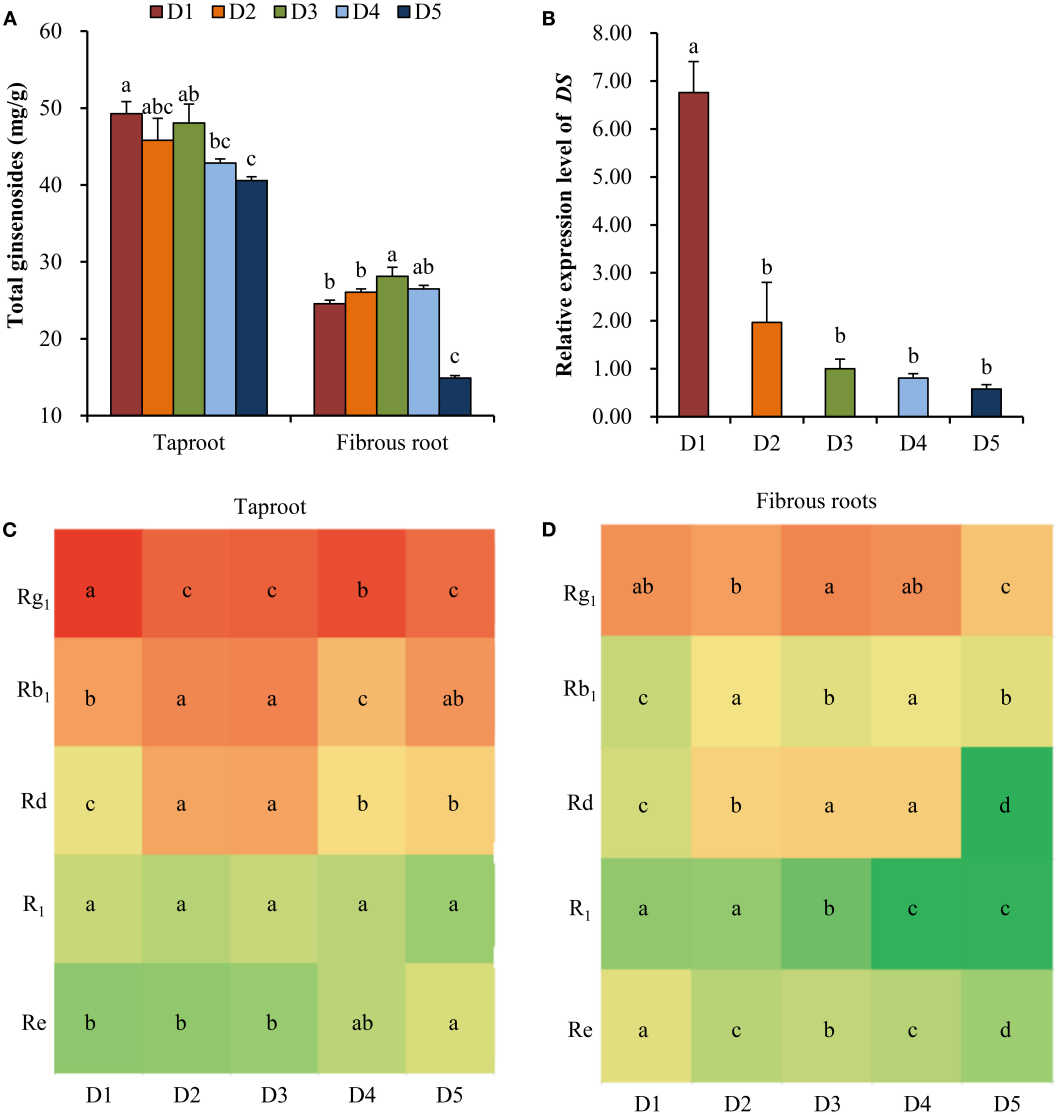
Effects of planting densities on the content of ginsenoside and relative expression level of *DS*. **(A)** Total contents of five ginsenosides in the taproot and fibrous root. **(B)** Relative expression level of *DS*. Monomeric ginsenoside in the taproot **(C)** and fibrous roots **(D)** of *P. notoginseng*. D1~D5 represent densities of 8 × 8, 10 × 10, 15 × 15, 20 × 20, and 30 × 30 cm, respectively. Error bars indicate the standard error (SE). Different letters indicate statistically significant differences among the five density treatments (*p* < 0.05; *n* = 4). The different colors in **(C)** and **(D)** indicate the changes in monomer ginsenoside content in taproots and fibrous roots.

At all planting densities, the total contents of five ginsenosides (Rg_1_, R_1_, Re, Rb_1_ and Rd) in taproot were high at densities D1 (8 × 8 cm), D2 (10 × 10 cm), and D3 (15 × 15 cm) compared with those at lower densities (Figure 3A). In the fibrous roots, the total contents of the five ginsenosides were significantly higher at density D3 (15 × 15 cm) than at high densities (8 × 8 and 10 × 10 cm) and at the lowest density (30 × 30 cm) (Figure 3A). Specifically, the 15 × 15 cm density showed greater accumulation of Rb_1_, Rd and R_1_ in taproots (Figure 3C) and Rg_1_ and Rd in fibrous roots (Figure 3D) than the other densities. The relative expression level of *DS* in fibrous roots increased with the increase of planting density (Figure 3B). Especially, the expression level of *DS* at the density of 8 × 8 cm was 6.76 times compared with the middle density of 15 × 15 cm (Figure 3B).

### Metabolic Profiling of Fibrous Roots at Different Plant Densities

**Figure 4:**
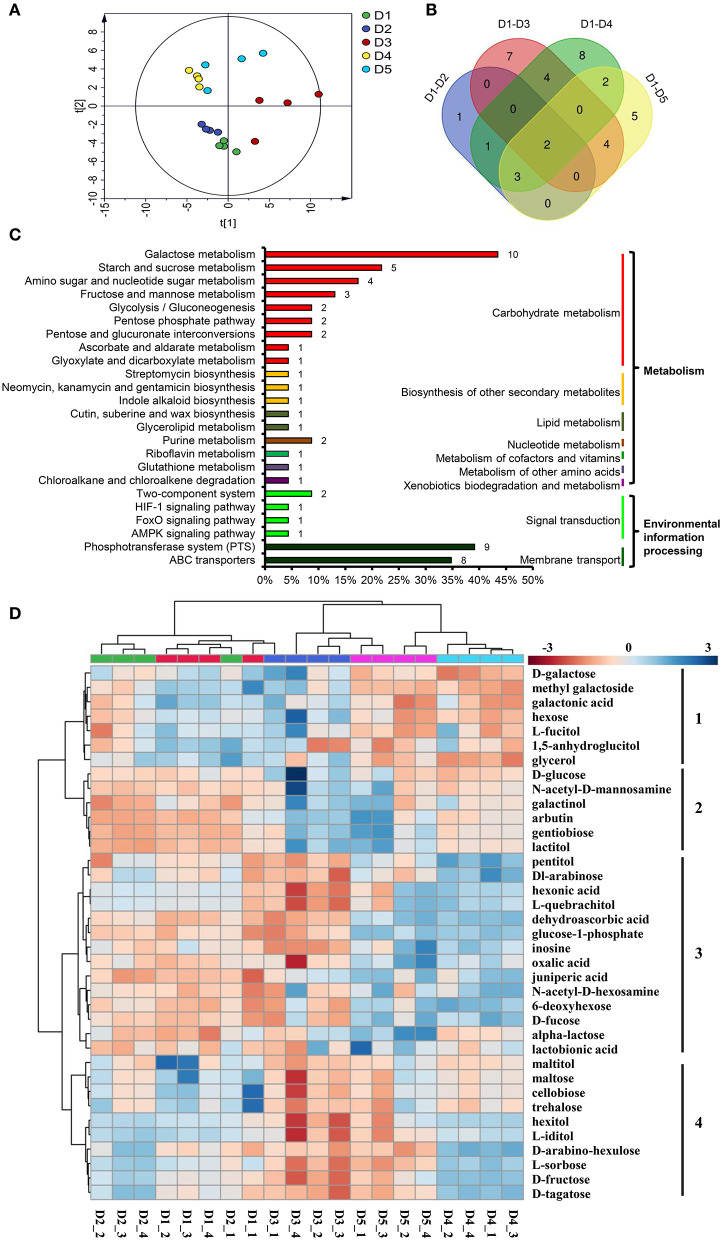
DAMs in the fibrous roots of *P. notoginseng* grown at different planting densities. **(A)** Score plot of the PCA model of all samples. **(B)** Venn diagram of the DAMs in four pairwise comparisons. **(C)** DAMs involved in metabolic pathways. The ordinate is the pathway name, and the colored line on the right indicates the pathway type. The number on the bar chart is the number of DAMs annotated in the pathway. The x-coordinate represents the proportion of the annotated DAMs in that pathway to all annotated DAMs. **(D)** Heat map comparing the relative intensity of DAMs among the different density treatments. The dendrogram on the left clusters similarly extracted metabolites based on hierarchical clustering, and the heat map displays the intensity of metabolites normalized within each row (metabolite). The dendrogram on the top indicates the clusters of the five density treatments. D1-D5 represent densities of 8 × 8, 10 × 10, 15 × 15, 20 × 20, and 30 × 30 cm, respectively.

The PCA separated the five density treatments into three groups. The two low densities, D4 (20 × 20 cm) and D5 (30 × 30 cm), were clustered into one group; the two high densities, D1 (8 × 8 cm) and D2 (10 × 10 cm), were clustered into another group; and the third group contained the moderate density, D3 (Figure 4A). OPLS-DA was used to identify the DAMs between D1 and D2, D1 and D3, D1 and D4, and D1 and D5 (Supplementary Table 5, Supplementary Figure 3). The results showed that 7, 17, 20 and 16 DAMs were identified in the four pairwise comparison groups (Supplementary Table 6). In total, there were 37 DAMs in the four pairwise comparisons (Figure 4B).

All 37 DAMs affected by planting density were mapped to their corresponding biological pathways in the KEGG. The 37 DAMs were assigned to 24 pathways. Of the 24 pathways, 18 were related to metabolism and 6 were related to environmental information processing (Figure 4C). The metabolic pathways significantly influenced by density were carbohydrate metabolism pathways, including galactose metabolism, starch and sucrose metabolism, amino sugar and nucleotide sugar metabolism, fructose and mannose metabolism. In addition, the biosynthesis of other secondary metabolites, including lipid metabolism, nucleotide metabolism, metabolism of cofactors and vitamins, metabolism of other amino acids, and xenobiotics biodegradation and metabolism, were affected. Pathways involved in environmental information processing, including signal transduction and membrane transport, were also enriched (Figure 4C).

A heat map was generated to reveal the changes in the contents of the 37 DAMs among the different planting densities. All DAMs could be classified into four clusters (Figure 4D). The DAMs in cluster 1 were enriched at the lower densities, D4 and D5, and are mainly involved in galactose metabolism (D-galactose, galactonic acid and glycerol). The DAMs in cluster 2, which showed higher accumulation at the higher densities, D1 and D2, mainly participate in glycolysis/gluconeogenesis (D-glucose and arbutin), galactose metabolism (D-glucose and galactinol), amino sugar and nucleotide sugar metabolism (D-glucose and N-acetyl-D-mannosamine) and the phosphotransferase system (D-glucose and arbutin). The DAMs in cluster 3 were increased at moderate and high densities, which were mainly involved in cutin, suberine and wax biosynthesis (juniperic acid); ascorbate and aldarate metabolism (dehydroascorbic acid); glutathione metabolism (dehydroascorbic acid); ABC transporters (pentitol, inosine and alpha-lactose); galactose metabolism (glucose-1-phosphate and alpha-lactose); amino sugar and nucleotide sugar metabolism (glucose-1-phosphate and 6-deoxyhexose); purine metabolism (inosine and oxalic acid). The DAMs grouped in cluster 4 were enriched at the moderate density, D3, and were mainly involved in carbohydrate metabolism, starch and sucrose metabolism (D-maltose, trehalose, D-fructose and D-maltose), galactose metabolism (hexitol, D-fructose and D-tagatose), fructose and mannose metabolism (L-sorbose and D-fructose); D-maltose, cellobiose and trehalose were also participated in ABC transporters and phosphotransferase system (Figure 4D).

### Correlation Analysis Between the Ginsenoside Content and Metabolites in *P. notoginseng* Roots

**Figure 5:**
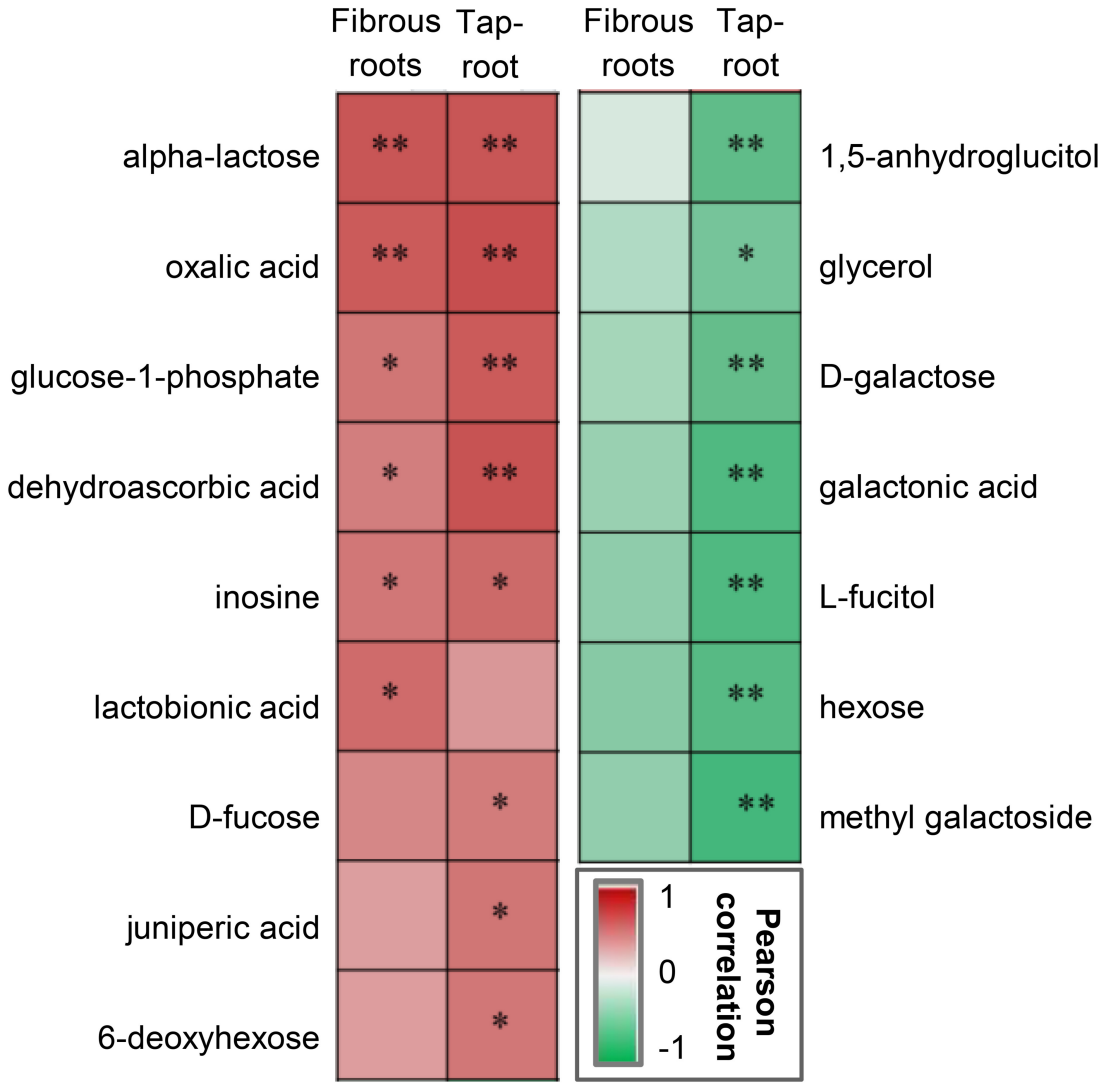
Pearson correlation analysis between DAMs and ginsenosides in the fibrous roots and taproots of *P. notoginseng*. *Indicates a significant correlation at *p* < 0.05 and **indicates a significant correlation at *p* < 0.01. The red box represents a positive correlation, the green box represents a negative correlation, and the depth of the color represents the degree of correlation.

Correlation analysis was conducted to determine whether there were correlations between ginsenoside accumulation and the DAMs (Figure 5). Some metabolites, including saccharides (alpha-lactose), saccharic acid (glucose-1-phosphate), organic acids (dehydroascorbic acid, oxalic acid), and alcohol (inosine), were significantly positively correlated with ginsenoside accumulation in both fibrous roots and taproots. In contrast, metabolites including sugars (D-galactose and hexose) and sugar-related metabolites (1,5-anhygroglucitol, glycerol, galactonic acid, L-fucitol and methyl galactoside), were significantly negatively correlated with the accumulation of ginsenosides in *P. notoginseng* taproots.

## Discussion

Medicinal herbs are special crops, their target products are secondary metabolites that are used as ingredients in herbal medicines. The key goal for the optimal production of herbal medicine is to simultaneously optimize the growth and content of secondary metabolites. Various genetic, ontogenic, morphogenetic, environmental factors, and agricultural practices can influence the biosynthesis of secondary metabolites (Yang et al., 2018a). Among them, adjusting the planting density is an effective agricultural practice that influences the growth and quality of crops by changing the amount of resources available to individual plants (Khan et al., 2018; Huang et al., 2019; Ning et al., 2019). In this study, we found that planting density could affect intraspecific interactions in *P. notoginseng* to modify the balance of the primary and secondary metabolism and consequently affect the growth and ginsenoside accumulation. The moderate density (15 × 15 cm) was better for the accumulation of dry biomass and ginsenosides than the high (8 × 8 and 10 × 10 cm) and low densities (20 × 20 and 30 × 30 cm). These findings suggest a way to optimize the growth and quality of herbal medicine by modifying planting density.

### Intraspecific Interactions Affected the Growth of *P. notoginseng*

In this study, we found that the plant height, root length, surface area, and root volume per plant were significantly higher at the highest planting density (8 × 8 cm) than at the other densities (Figures 1C,E–G). Plant morphological parameters such as plant height and root system are significantly influenced by the planting density (Khan et al., 2018; Luo et al., 2018). At a high planting density, the high intraspecific competition will result in changes to plant organ morphology that facilitate the uptake and utilization of light, water and nutrients to ensure normal plant development (Ning et al., 2019). It is remarkable that a high planting density has adverse effects on plants due to the intense intraspecific competition for resources. Previous study demonstrated that high density decreased the light transmittance to low layer of plant population and reduced yield (Dong et al., 2006). At the middle planting density of 15 × 15 cm, the dry biomass of *P. notoginseng* per plant was highest compared with other planting densities (Figure 1D). This data was consistent with previous studies that 10~15 × 12~20 cm was the commonly used planting density of *P. notoginseng* and could achieved the high yield and economic benefits (Cui et al., 1998; Zheng et al., 2015; Ou et al., 2018; Li et al., 2020). Interestingly, the plant height, dry biomass, root length, surface area and root volume were all significantly decreased at the lowest planting density (30 × 30 cm), in which the resources were relatively sufficient, compared with those at the higher planting densities. Previous literature shows that a positive interaction formed during long-term evolution exists in plant populations, which will alleviate population competition and improve resource utilization (Huang et al., 2019). Some further research suggested that trace quantities of small-molecular substances may be involved in this positive interaction (Dorokhov et al., 2018; Takabayashi and Shiojiri, 2019). Therefore, we speculated that the positive interaction may be occurred between individual *P. notoginseng* plants at appropriate planting density and promoted the plant growth. When the distance between plants increases, such as the lowest density of 30 × 30 cm, this communication will weaken or even disappear, and some growth indicators (height, root morphology, etc.) were reduced. Of course, which substances play important roles in these interactions and how *P. notoginseng* collaborate still need to be further researched.

### Intraspecific Interactions Affected the Metabolism of *P. notoginseng*

Plants have coevolved to adapt to stress conditions in order to maintain their normal physiology and survive (Shepherd and Griffiths, 2006). A previous study demonstrated that poplar (*Populus* × *euramericana*) could change its metabolism to adapt to crowding stress under high-density conditions (Ning et al., 2019). The typically utilized medicinal part of *P. notoginseng* is the root (Cao et al., 2019), and the fibrous roots are more susceptible to environmental stress than the taproot (Veronica et al., 2018). Thus, it is meaningful to focus on the metabolism of fibrous roots in response to different planting densities. Based on the metabolomics analysis, the five planting densities could be divided into three groups: the high densities (8 × 8 and 10 × 10 cm), the intermediate density (15 × 15 cm) and the low densities (20 × 20 and 30 × 30 cm) (Figure 4A). These groupings indicated that the intraspecific interactions affected the metabolism of *P. notoginseng*.

Previous reports have found that stress conditions can induce the burst of reactive oxygen species (ROS) (Jorge et al., 2016). In this study, intraspecific competition among *P. notoginseng* at the high planting densities caused environmental stress, and the relative content of the antioxidants gentiobiose (Yang et al., 2018b), dehydroascorbic acid (Dewhirst and Fry, 2018), and oxalic acid (Martínez-Esplá et al., 2019) were significantly increased at the high planting densities (Figure 4D). These increased metabolites may be involved in eliminating excessive ROS. Other metabolites associated with plant stress, such as juniperic acid involved in cutin, suberine and wax biosynthesis, accumulated significantly at the high densities of 8 × 8 and 10 × 10 cm (Figure 4D). The biosynthetic pathway of cutin and waxes is sensitive to environmental stresses; strengthening the cuticular wax layer of the plant provides a better protective barrier against a wide range of abiotic stresses (Shepherd and Griffiths, 2006). Phytohormones, which are special plant metabolites, are the essential endogenous molecules to regulate plant growth and response to diverse stresses (Ryu and Cho, 2015). Changes in planting density also caused content changes of some plant hormones, such as SA, JA and IAA (Figure 2). SA and JA play pivotal roles in the regulation of plant biotic and abiotic stress responses (Kazan, 2015; Rekhter et al., 2019; Wassie et al., 2020). Previous study demonstrated that plants could balance their growth and defense responses through JA signaling pathway under shade condition induced by high-density planting (Liu et al., 2019); SA affect the antioxidative metabolism and modulate cellular redox homeostasis (Janda et al., 2020). Here, the contents of SA and JA in roots significantly increased at the highest density, which could be an adaptive response to better adapt to environmental and resource stresses caused by intraspecific competition.

Allocation theory assumes that plants need to divide the limited essential resources among different competing physiological functions, including growth, maintenance, reproduction, defense, etc. (Caretto et al., 2015). Moderate intraspecific competition at the planting density of 15 × 15 cm did not need to consume excessive resources to alleviate the damage caused by excessive stress. Metabolomics analysis confirmed that some metabolites (including maltose, cellobiose, trehalose, and D-fructose) involved in starch and sucrose metabolism were all significantly up-regulated at density 15 × 15 cm. Starch is the major carbohydrate storage molecule in plants and is synthesized from transported sucrose (Thalmann and Santelia, 2017). The enhancement of this metabolic pathway allows plants to store more carbohydrates. Moreover, the increase of growth-promoting hormone IAA (Figure 2C) also indicated that 15 × 15 cm was more suitable for the growth of *P. notoginseng*. Therefore, middle density of 15 × 15 cm with moderate environmental stress obtained the highest dry biomass of *P. notoginseng* rather than 8 × 8 cm with the strongest environmental stress (Figure 1D). Surprisingly, the low planting densities (20 × 20 and 30 × 30 cm), which were able to supply enough resources for plant growth, produced the lowest plant height and dry biomass (Figures 1C,D). The production of some metabolites participated in galactose metabolism (D-galactose, galactonic acid and glycerol) were significantly increased at low densities (Figure 4D). D-galactose is an important constituent of plant cell walls and sugars such as stachyose and raffinose (Roberts et al., 1971; Ng et al., 2015). However, a previous study demonstrated that exogenous D-galactose was toxic and could inhibit the growth of many plant tissues, including roots, coleoptiles and germinating pollen, above certain concentrations (Roberts et al., 1971; Ryoichi and Yoshio, 1984). Therefore, the significant accumulation of D-galactose and related metabolites may be the reason for the poor growth of *P. notoginseng* at low densities in terms of plant metabolism.

### Intraspecific Interactions Affected the Synthesis of Ginsenosides

Medicinal herbs are known to produce a wide variety of therapeutic secondary metabolites, and moderate environmental stress and necessary available resources are important for the synthesis of secondary metabolites to produce high-quality, genuine medicinal materials (Guo et al., 2020). Ginsenoside is the major secondary metabolite in ginseng plants and has numerous physiological and pharmacological effects (Liu et al., 2017b). In this study, the high content of ginsenosides in taproot and fibrous root from high (8 × 8, 10 × 10 cm) to moderate (15 × 15 cm) densities (Figure 3A) and the high *DS* expression with the increase of planting density (Figure 3B) demonstrated that high intensity of intraspecific interactions enhanced the synthesis of ginsenosides. In addition, previous study has found that high level of SA and JA could promote the biosynthesis of ginsenoside (Wang et al., 2016). Here, the high contents of SA and JA at density of 8 × 8 cm (Figures 2A,B) confirmed the high intraspecific interaction and high level of ginsenoside synthesis. A growing number of studies have suggested that synthesis and accumulation of secondary metabolites are the most important defense strategies of medicinal plants against environmental stress (Guo et al., 2020). However, excessive environmental stress will not only reduce the yield production, but also reduce the accumulation of secondary metabolites in medicinal plants (Huang and Guo, 2007). It has been documented that primary metabolism provides necessary energy and precursor substances for the biosynthesis of secondary metabolites (Ramakrishna and Ravishankar, 2011; Caretto et al., 2015; Liu et al., 2017a). In the ginsenoside synthesis pathway, primary metabolites are connected with ginsenoside accumulation (Kim et al., 2015; Chen et al., 2017). In this study, the correlation analysis found that some metabolites, such as glucose-1-phosphate, oxalic acid, dehydroascorbic acid etc., were significantly positively correlated with the accumulation of total ginsenosides in fibrous roots and taproots (Figure 5). The relative contents of these metabolites were higher at high and middle planting densities (Figure 4D). Glucose-1-phosphate is a precursor of UDP-glucose, and UDP-glucose acts as the substrate that binds to the sapogenin catalyzed by UDP glycosyltransferase (UGT) to produce ginsenosides (Benini et al., 2017; Upadhyay et al., 2018). Therefore, an increase in glucose-1-phosphate levels may directly promote the synthesis of ginsenosides at high and moderate densities. In addition, some sugars involved in starch and sucrose metabolism, including cellobiose, maltose, trehalose, and D-fructose, were all significantly upregulated at the moderate density of 15 × 15 cm (Figure 4D), which indicated that more carbon was fixed at the suitable planting density. Previous studies have shown that high rates of carbon assimilation to carbon accumulation are closely associated with ginsenoside accumulation in *P. ginseng* taproots and *P. quinquefolius* lateral roots (Liu et al., 2017a,b). Therefore, moderate environmental stress at the density of 15 × 15 cm promoted the plant growth and the synthesis of secondary metabolites through balancing primary metabolism and secondary metabolism, resulting in high dry biomass and ginsenoside accumulation simultaneously.

DAMs, such as 1,5-anhydroglucitol, glycerol, D-galactose, galactonic acid, L-fucitol, hexose, and methyl galactoside, were negatively correlated with the total ginsenoside content of *P. notoginseng* taproots (Figure 5), and the contents of these DAMs were upregulated at low densities (20 × 20 and 30 × 30 cm) (Figure 4D). Three of these metabolites, glycerol, D-galactose and galactonic acid, participated in the galactose metabolism pathway. Previous studies demonstrated that excessive D-galactose caused poor plant growth; furthermore, abnormal metabolite accumulation may interfere with normal carbohydrate metabolism (Roberts et al., 1971; Ryoichi and Yoshio, 1984). Therefore, the increase in the contents of D-galactose and related metabolites was not beneficial to the accumulation of ginsenosides, and this increase may be closely related to abnormal carbohydrate metabolism in *P. notoginseng*.

## Conclusion

Overall, an appropriate planting density (15 × 15 cm) had a beneficial effect on plant growth and ginsenoside accumulation in *P. notoginseng* by balancing primary metabolism and secondary metabolism. Adjusting the planting density is an effective way to optimize growth and ginsenoside production in *P. notoginseng*.

## Data Availability Statement

The original contributions presented in the study are publicly available. This data can be found here: www.ebi.ac.uk/metabolights/MTBLS2634, the accession number is MTBLS2634.

## Author Contributions

MY and SZ conceived the ideas and directed the project. HL and HG performed the field experiment and collected the relevant data. CY, CG, and YZ performed GC-MS and HPLC-MS/MS analysis. MY, SZ, HL, and HG wrote the manuscript. HH, YL, and XH improved it. All authors contributed to the article and approved the submitted version.

## Conflict of Interest

The authors declare that the research was conducted in the absence of any commercial or financial relationships that could be construed as a potential conflict of interest.

## Supplementary Material

Supplementary Figure 1 UPLC chromatograms of ginsenosides in mixed standards, taproot, and fibrous root.

Supplementary Figure 2 GC-MS chromatograms of internal standard ribitol among different planting densities.

Supplementary Figure 3 Score plot of OPLS-DA model and two hundred permutations were performed, and R^2^ and Q^2^ values were plotted. Green triangle: R^2^. blue square: Q^2^. The green line represents the regression line for R^2^ and the blue line for Q^2^. Pairwise comparison included D1 and D2 **(A,B)**, D1 and D3 **(C,D)**, D1 and D4 **(E,F)**, D1 and D5 **(G,H)**. D1-D5 represent the density of 8 × 8, 10 × 10, 15 × 15, 20 × 20, and 30 × 30 cm, respectively.

Supplementary Table 1 Solvent system of ginsenosides analysis by UPLC.

Supplementary Table 2 Primers used in the *DS* and *18S* gene expression analysis.

Supplementary Table 3 The identification parameters of MS-DIAL.

Supplementary Table 4 The raw MS-DIAL output of the metabolomics.

Supplementary Table 5 Explanation rate of cumulative model.

Supplementary Table 6 DAMs in five planting densities.
