# Supplementary figures and images for: Planting Density Affects Panax notoginseng Growth and Ginsenoside Accumulation by Balancing Primary and Secondary Metabolism
Source: Front Plant Sci. 2021 Apr 12;12:628294. doi: 10.3389/fpls.2021.628294 (PMC8086637; doi:10.3389/fpls.2021.628294)

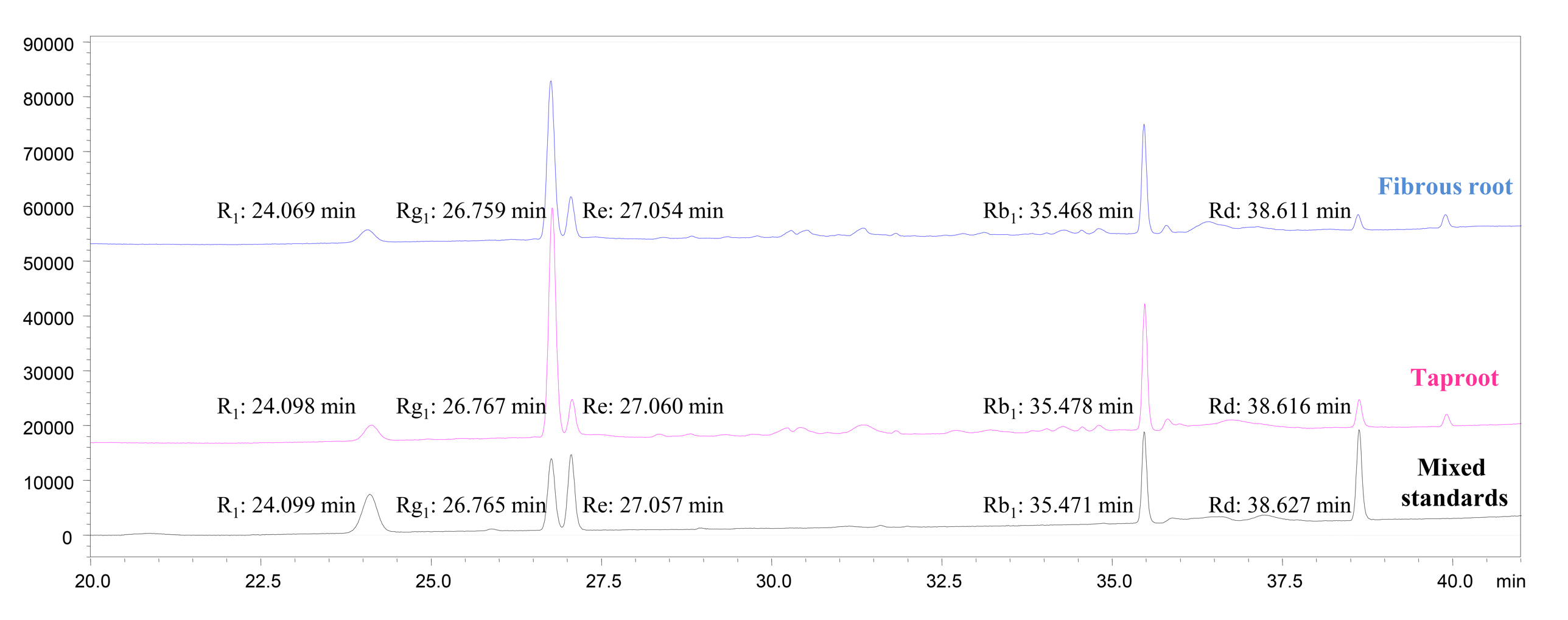

Supplement: Supplementary Figure 1 — UPLC chromatograms of ginsenosides in mixed standards, taproot, and fibrous root. [file Image_1.TIF]

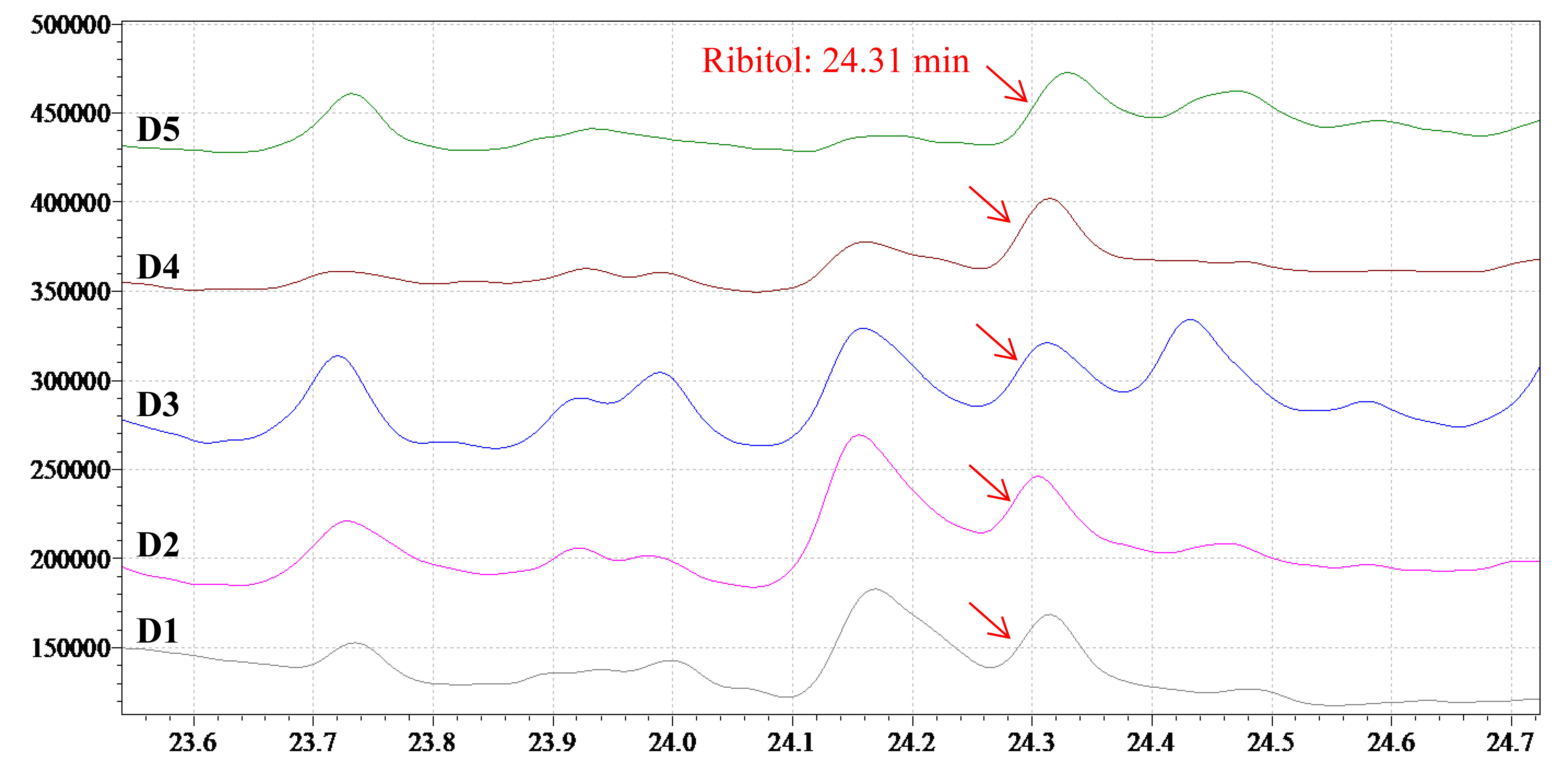

Supplement: Supplementary Figure 2 — GC-MS chromatograms of internal standard ribitol among different planting densities. [file Image_2.TIF]

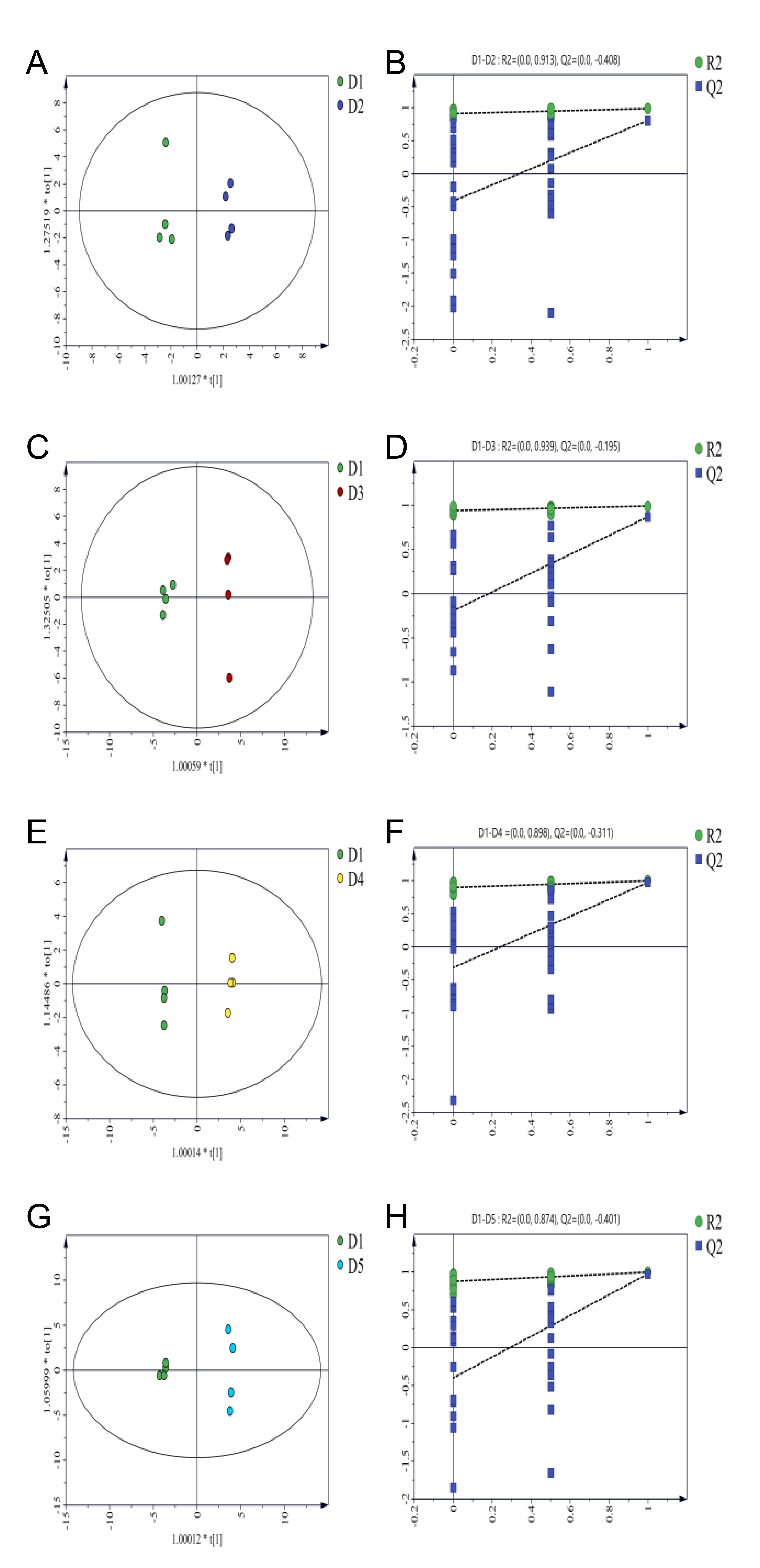

Supplement: Supplementary Figure 3 — Score plot of OPLS-DA model and two hundred permutations were performed, and R2 and Q2 values were plotted. Green triangle: R2. blue square: Q2. The green line represents the regression line for R2 and the blue line for Q2. Pairwise comparison included D1 and D2 (A,B), D1 and D3 (C,D), D1 and D4 (E,F), D1 and D5 (G,H). D1-D5 represent the density of 8 × 8, 10 × 10, 15 × 15, 20 × 20, and 30 × 30 cm, respectively. [file Image_3.TIF]
