# Supplementary material for: Planting Density Affects Panax notoginseng Growth and Ginsenoside Accumulation by Balancing Primary and Secondary Metabolism
Source: Front Plant Sci. 2021 Apr 12;12:628294. doi: 10.3389/fpls.2021.628294 (PMC8086637; doi:10.3389/fpls.2021.628294)
Supplement: Supplementary Table 1 — Solvent system of ginsenosides analysis by UPLC. [file Table_1.DOCX]

Table S1 Solvent system of ginsenosides analysis by UPLC

| Stage | Time | Solvent A |
| --- | --- | --- |
| 1 | 0~20.0 min | 1%~18% |
| 2 | 20.0~40.0 min | 18%~40% |
| 3 | 40.0~45.0 min | 40%~55% |
| 4 | 45.0~50.0 min | 55% |
| 5 | 50.0~51.0 min | 55%~18% |
