# Supplementary material for: Planting Density Affects Panax notoginseng Growth and Ginsenoside Accumulation by Balancing Primary and Secondary Metabolism
Source: Front Plant Sci. 2021 Apr 12;12:628294. doi: 10.3389/fpls.2021.628294 (PMC8086637; doi:10.3389/fpls.2021.628294)
Supplement: Supplementary Table 2 — Primers used in the DS and 18S gene expression analysis. [file Table_2.DOCX]

Table S2 Primers used in the *DS* and *18S* gene expression analysis

| Primer ID | Squence (5´-3´) |
| --- | --- |
| *DS*-FP | CGAGAAATTTACACCACTCAAG |
| *DS*-RP | CCATTATCCATTTGCGCATTG |
| Pn-*18S* FP | GATGCGCTCCTGTCCTTAAC |
| Pn-*18S* RP | CATCCTTGGCAAATGCTTTC |
