# Supplementary material for: Planting Density Affects Panax notoginseng Growth and Ginsenoside Accumulation by Balancing Primary and Secondary Metabolism
Source: Front Plant Sci. 2021 Apr 12;12:628294. doi: 10.3389/fpls.2021.628294 (PMC8086637; doi:10.3389/fpls.2021.628294)
Supplement: Supplementary Table 3 — The identification parameters of MS-DIAL. [file Table_3.DOCX]

Table S3 The identification parameters of MS-DIAL

| Step | Measures | Analysis Parameter Setting |
| --- | --- | --- |
| 1 | Data collection | a. *Mass scan range*: begin from 85 Da, end of 500 Da  b. Advanced: rention time begin from 0 to 100 min  c. *Multithreading*: Number of threads is 1 |
| 2 | Peak detection | a. *Peak detection parameters*: Minimum peak height is 5000 amplitude |
| 3 | Ms1Dec | a. *Deconvolution parameters*: Sigma window value is 0.5; EI spectra cut off is 1 |
| 4 | Identification | a. *Retention time setting*:  RI or RT: Use retention index (RI);  Index file: set (a txt. file containing the retention time of 13 saturated fatty acid methyl esters: C8, C9, C10, C12, C14, C16, C18, C20, C22, C24, C26, C28, C30);  Index type: FAMES;  b. *MSP file and identification setting*:  MSP file: Feihn library;  Retention index tolerance: 10000;  m/z tolerance: 0.5 Da;  EI similarity cut off: 70%;  Identification score cut off: 70%;  Use retention information for scoring: yes;  Use quant masses defined in MSP format file: yes |
| 5 | Alignment | a. *Parameter setting*:  RI or RT: Use retention time (min);  Retention time tolerance : 0.1min;  EI similarity tolerance: 70%  b. Advanced:  Gap filling by compulsion: yes |
| 6 | Filtering | a. *Filtering option for alignment result*:  Detected in all QCs: no;  Remove features based on blank information: yes;  Sample max/ blank average: 3 fold change |
| Note: Parameters not mentioned in the above table were default parameters. | | |
