# Supplementary material for: Planting Density Affects Panax notoginseng Growth and Ginsenoside Accumulation by Balancing Primary and Secondary Metabolism
Source: Front Plant Sci. 2021 Apr 12;12:628294. doi: 10.3389/fpls.2021.628294 (PMC8086637; doi:10.3389/fpls.2021.628294)
Supplement: Supplementary Table 5 — Explanation rate of cumulative model. [file Table_5.DOCX]

Table S5 Explanation rate of cumulative model

| Comparative groups | R^2^X(cum) | R^2^Y(cum) | Q^2^(cum) |
| --- | --- | --- | --- |
| D1-D2 | 0.753 | 0.988 | 0.809 |
| D1-D3 | 0.784 | 0.991 | 0.87 |
| D1-D4 | 0.839 | 0.998 | 0.974 |
| D1-D5 | 0.853 | 0.996 | 0.974 |
