# Supplementary material for: Planting Density Affects Panax notoginseng Growth and Ginsenoside Accumulation by Balancing Primary and Secondary Metabolism
Source: Front Plant Sci. 2021 Apr 12;12:628294. doi: 10.3389/fpls.2021.628294 (PMC8086637; doi:10.3389/fpls.2021.628294)
Supplement: Supplementary Table 6 — DAMs in five planting densities. [file Table_6.DOCX]

Table S6 DAMs in five planting densities

| Comparison groups | Peak | VIP | p value | Fold change |
| --- | --- | --- | --- | --- |
| D1-D2 | down-regulated |  |  |  |
|  | dehydroascorbic acid | 1.76 | 0.0004 | 2.06 |
|  | up-regulated |  |  |  |
|  | galactonic acid | 1.27 | 0.0269 | 1.26 |
|  | hexose | 1.22 | 0.0345 | 1.47 |
|  | galactinol | 1.58 | 0.0032 | 1.50 |
|  | maltose | 1.49 | 0.0353 | 1.57 |
|  | methyl galactoside | 1.77 | 0.0179 | 2.03 |
|  | cellobiose | 1.70 | 0.0305 | 2.10 |
| D1-D3 | down-regulated |  |  |  |
|  | arbutin | 1.51 | 0.0069 | 3.32 |
|  | lactitol | 1.38 | 0.0095 | 2.28 |
|  | N-acetyl-D-mannosamine | 1.35 | 0.0214 | 2.13 |
|  | gentiobiose | 1.26 | 0.0175 | 2.04 |
|  | juniperic acid | 1.18 | 0.0283 | 1.77 |
|  | D-glucose | 1.25 | 0.0307 | 1.71 |
|  | up-regulated |  |  |  |
|  | L-sorbose | 1.23 | 0.0416 | 1.44 |
|  | D-fructose | 1.20 | 0.0289 | 1.49 |
|  | inosine | 1.28 | 0.0112 | 1.85 |
|  | trehalose | 1.50 | 0.0028 | 1.86 |
|  | L-quebrachitol | 1.31 | 0.0288 | 2.15 |
|  | maltose | 1.65 | 0.0017 | 2.27 |
|  | maltitol | 1.63 | 0.0138 | 2.30 |
|  | L-iditol | 1.44 | 0.0390 | 2.52 |
|  | hexonic acid | 1.42 | 0.0177 | 2.75 |
|  | cellobiose | 1.88 | 0.0118 | 4.28 |
|  | hexitol | 1.65 | 0.0422 | 4.95 |
| D1-D4 | down-regulated |  |  |  |
|  | dehydroascorbic acid | 1.99 | 0.0000 | 11.95 |
|  | Dl-arabinose | 1.61 | 0.0093 | 3.56 |
|  | adonitol | 1.63 | 0.0081 | 3.54 |
|  | glucose-1-phosphate | 1.45 | 0.0084 | 2.50 |
|  | juniperic acid | 1.31 | 0.0201 | 2.00 |
|  | inosine | 1.09 | 0.0166 | 1.95 |
|  | 6-deoxyhexose | 1.42 | 0.0002 | 1.81 |
|  | D-fructose | 1.13 | 0.0009 | 1.55 |
|  | N-acetyl-D-hexosamine | 1.17 | 0.0205 | 1.54 |
|  | D-fucose | 1.18 | 0.0041 | 1.46 |
|  | D-fructofuranose | 1.14 | 0.0074 | 1.41 |
|  | D-tagatose | 1.03 | 0.0115 | 1.35 |
|  | up-regulated |  |  |  |
|  | galactonic acid | 1.06 | 0.0053 | 1.33 |
|  | D-galactose | 1.21 | 0.0071 | 1.49 |
|  | trehalose | 1.11 | 0.0161 | 1.66 |
|  | maltose | 1.11 | 0.0081 | 1.67 |
|  | hexose | 1.18 | 0.0017 | 1.78 |
|  | glycerol | 1.47 | 0.0002 | 2.17 |
|  | cellobiose | 1.28 | 0.0066 | 2.33 |
|  | methyl galactoside | 1.64 | 0.0001 | 3.16 |
| D1-D5 | down-regulated |  |  |  |
|  | dehydroascorbic acid | 1.85 | 0.0000 | 7.11 |
|  | glucose-1-phosphate | 1.45 | 0.0261 | 2.06 |
|  | gentiobiose | 1.43 | 0.0264 | 1.96 |
|  | lactobionic acid | 1.27 | 0.0229 | 1.90 |
|  | lactitol | 1.36 | 0.0241 | 1.89 |
|  | alpha-lactose | 1.26 | 0.0080 | 1.31 |
|  | oxalic acid | 1.16 | 0.0052 | 1.30 |
|  | up-regulated |  |  |  |
|  | galactonic acid | 1.12 | 0.0324 | 1.63 |
|  | L-sorbose | 1.05 | 0.0012 | 1.65 |
|  | 1,5-anhydroglucitol | 1.09 | 0.0094 | 1.76 |
|  | maltose | 1.02 | 0.0096 | 1.96 |
|  | glycerol | 1.15 | 0.0076 | 1.98 |
|  | L-iditol | 1.13 | 0.0078 | 2.15 |
|  | L-fucitol | 1.40 | 0.0134 | 3.01 |
|  | cellobiose | 1.28 | 0.0158 | 3.04 |
|  | methyl galactoside | 1.60 | 0.0002 | 3.44 |
